# *Mycobacterium abscessus* biofilms produce an extracellular matrix and have a distinct mycolic acid profile

**DOI:** 10.1016/j.tcsw.2021.100051

**Published:** 2021-04-06

**Authors:** Anja Dokic, Eliza Peterson, Mario L. Arrieta-Ortiz, Min Pan, Alessandro Di Maio, Nitin Baliga, Apoorva Bhatt

**Affiliations:** a School of Biosciences and Institute of Microbiology and Infection, University of Birmingham, Edgbaston, Birmingham B15 2TT, United Kingdom; b Institute for Systems Biology, Seattle, WA 98109 USA

**Keywords:** *Mycobacterium abscessus*, Biofilm, Mycolic acid, Transcription, Extracellular matrix, Lipids, NTMs, Non-tuberculous mycobacteria, ECM, Extracellular matrix, SEM, Scanning electron microscopy, DEG, Differentially expressed genes, eDNA, Extracellular DNA, TLC, thin layer chromatography, MAMEs, mycolic acids as methyl esters

## Abstract

A non-tuberculous mycobacterium, *Mycobacterium abscessus* is an emerging opportunistic pathogen associated with difficult to treat pulmonary infections, particularly in patients suffering from cystic fibrosis. It is capable of forming biofilms *in vitro* that result in an increase of already high levels of antibiotic resistance in this bacterium. Evidence that *M. abscessus* forms biofilm-like microcolonies in patient lungs and on medical devices further implicated the need to investigate this biofilm in detail. Therefore, in this study we characterized the *M. abscessus* pellicular biofilm, formed on a liquid–air interface, by studying its molecular composition, and its transcriptional profile in comparison to planktonic cells. Using scanning electron micrographs and fluorescence microscopy, we showed that *M. abscessus* biofilms produce an extracellular matrix composed of lipids, proteins, carbohydrates and extracellular DNA. Transcriptomic analysis of biofilms revealed an upregulation of pathways involved in the glyoxylate shunt, redox metabolism and mycolic acid biosynthesis. Genes involved in elongation and desaturation of mycolic acids were highly upregulated in biofilms and, mirroring those findings, biochemical analysis of mycolates revealed molecular changes and an increase in mycolic acid chain length. Together these results give us an insight into the complex structure of *M. abscessus* biofilms, the understanding of which may be adapted for clinical use in treatment of biofilm infections, including strategies for dispersing the extracellular matrix, allowing antibiotics to gain access to bacteria within the biofilm.

## Introduction

Non-tuberculous mycobacteria (NTM) belong to the genus *Mycobacterium* and are environmental bacteria capable of causing disease in immunocompromised individuals. NTMs cause skin and soft tissue infections, lymphadenitis and aseptic meningitis, however most commonly they manifest as pulmonary infections (Faria et al., 2015, Ratnatunga et al., 2020). Since the discovery of the association of *Mycobacterium avium* infections with mortality in HIV patients, disseminated and mixed culture NTM infections were found in other immunocompromised populations suffering from chronic pulmonary disease, renal failure, cystic fibrosis (CF), leukaemia and in transplant recipients (Sousa et al., 2015).

*Mycobaterium abscessus* is a rapidly growing NTM and an emerging opportunistic pathogen most often causing chronic pulmonary disease in patients with underlying lung conditions (Medjahed et al., 2010). CF patients are particularly vulnerable, and it is estimated that 3–10% of CF patients in the USA and Europe are infected with *M. abscessus*, which can either result in a poor clinical outcome or life-long persistent infection without symptoms (Cystic Fibrosis Foundation, 2010). *M. abscessus* is the most commonly isolated rapidly growing mycobacteria from lung infections, which is alarming given the average rate of treatment success is only 45.6% (Kim et al., 2016, Kwak et al., 2019). None of the currently available treatments are curative or have been shown as effective in long-term sputum conversion in patients with chronic lung disease (Kwak et al., 2019).

A key strategy of NTM pathogenesis is the ability to form biofilms in the environment as well as in water distribution systems and on hospital equipment (Galassi et al., 2003, Ghosh et al., 2017, Zambrano and Kolter, 2005). Notably, *Mycobacterium fortuitum*, *Mycobacterium chelonae* and *M. abscessus* subsp. *massiliense* have all been associated with biofilm formation on medical implants (Bosio et al., 2012, Padilla et al., 2018, Rüegg et al., 2015). Biofilms are communities of aggregated bacteria that form in response to stress and as a part of this survival strategy bacteria within the biofilm undergo genetic and metabolic changes (Chakraborty and Kumar, 2019, Solokhina et al., 2017, Vasudevan, 2014). In the host, biofilms contribute to virulence by mediating colonization, evading the host’s immune response and limiting diffusion of antimicrobials (Aung et al., 2017, Faria et al., 2015, Roux et al., 2016).

Bacteria in biofilms are enveloped in an extracellular matrix (ECM) which is required for cell aggregation, adhesion to surfaces, retention of water and nutrient provision. The ECM also serves as a physical barrier, allowing bacteria a significant degree of resistance to water treatment, disinfectants and antibiotics (Aung et al., 2016, Aung et al., 2017, Flemming and Wingender, 2010, Galassi et al., 2003, Greendyke and Byrd, 2008). Secreted components that constitute an ECM can be lipids, carbohydrates, proteins or extracellular DNA (eDNA) (Chakraborty and Kumar, 2019, Flemming and Wingender, 2010, Flemming et al., 2016, Karygianni et al., 2020, Vega-Dominguez et al., 2020). In mycobacterial biofilms, lipids, particularly mycolyl-diacylglycerol, mycolic acids and glycopeptidolipids (Chen et al., 2006, Ojha et al., 2005, Recht and Kolter, 2001, Yang et al., 2017), are important for biofilm formation and, additionally, strains defective in exporting cell wall lipids have a defect in biofilm formation ( Pacheco et al., 2013). Biofilms of *Mycobacterium tuberculosis* and *M. avium* have been demonstrated to form a biofilm ECM (Rose et al., 2015, Trivedi et al., 2016), and we have recently shown the same for *M. chelonae* (Vega-Dominguez et al., 2020). An earlier study found that carbohydrates are the major component of the extracellular material of *M. avium*, *Mycobacterium gastri* and *Mycobacterium kansasii*, while in *Mycobacterium smegmatis* and *Mycobacterium phlei* carbohydrates are less abundant in comparison to proteins (Lemassu et al., 1996). Carbohydrates are major components of *M. tuberculosis* and *M. chelonae* biofilm ECMs (Lemassu et al., 1996, Trivedi et al., 2016, Vega-Dominguez et al., 2020) and while they seem to play a structural role, the role of proteins has not been fully characterized but there is evidence that treatment with Proteinase K reduces overall biofilm biomass in *M. tuberculosis* (Trivedi et al., 2016). eDNA, identified in biofilms of *M. tuberculosis, M. avium, M. abscessus*, *M. fortuitum* and *M. chelonae* (Aung et al., 2017, Rose et al., 2015, Rose and Bermudez, 2016, Toyofuku et al., 2016, Trivedi et al., 2016, Vega-Dominguez et al., 2020, Whitchurch et al., 2002), plays a fundamental structural role by promoting attachment to surfaces and facilitating bacterial aggregation (Ibáñez de Aldecoa et al., 2017, Pakkulnan et al., 2019, Rose et al., 2015) as biofilms treated with DNase become less structurally secure and are more vulnerable to antibiotics (Cavaliere et al., 2014, Rose et al., 2015, Tetz et al., 2009, Trivedi et al., 2016).

Biofilms are suspected to play an important role in *M. abscessus* infections and there is evidence of biofilm-like microcolony formation in patient lungs (Fennelly et al., 2016, Qvist et al., 2015, Qvist et al., 2013). It has been demonstrated that *M. abscessus* has a decreased susceptibility to several first-line antibiotics, such as amikacin, cefoxitin, and clarithromycin, when grown in a biofilm *in vitro* (Greendyke and Byrd, 2008, Marrakchi et al., 2014). Previous research suggests that *M. abscessus* smooth colony variants colonize abnormal lung airways in a biofilm, and the spontaneous loss of surface glycopeptidolipids leads to a morphological change to the invasive rough colony type, causing inflammation and invasive disease (Howard et al., 2006, Rhoades et al., 2009). *M. abscessus* rough colony type is known to form pellicular biofilms, despite the lack of glycopeptidolipids (Clary et al., 2018), which have a higher degree of mechanical resistance compared to biofilms made by smooth colony types (Gloag et al., 2021) , but otherwise the composition of their ECMs has not been thoroughly studied.

Studies in *M. avium, M tuberculosis*, *M. smegmatis* and *M. chelonae* showed that mycobacterial biofilms are rich in carbohydrates, lipids, proteins and eDNA and have an altered transcription profile (Chakraborty and Kumar, 2019, Rose et al., 2015, Trivedi et al., 2016, Vega-Dominguez et al., 2020), however the same has not yet been shown in *M. abscessus*. In this study we focused on understanding biofilm formation in a smooth strain of *M. abscessus*, using a pellicular biofilm formed on a liquid–air interface as a model. Following a similar approach used for *M. chelonae* (Vega-Dominguez et al., 2020), utilising confocal laser scanning microscopy, scanning electron microscopy, transcriptomic and biochemical analysis, we have defined the structure, content and expression profiles of *M. abscessus* biofilms. A greater understanding of molecular events that drive biofilm formation and biochemical changes that occur in the cells is needed in order to improve the current strategies for combating *M. abscessus* infections.

## Materials and methods

### Culture conditions

*M. abscessus* ATCC 19977 Type strain was used for all experiments. Planktonic bacteria were grown in Sauton’s minimal media supplemented with 0.05% Tyloxapol shaking (180 rpm) at 37 °C. For transcriptomic analysis 1 L of culture was grown per timepoint, planktonic t1 was harvested 24 h post inoculation and planktonic t2 at 48 h. For biofilm formation, *M. abscessus* colonies grown on 7H11/OADC were resuspended in sterile 1xPBS and diluted to OD_600_ 0.05 in Sauton’s minimal media and grown stationary at 37 °C in a 24-well plate (for lipid analysis and microscopy) or in 75 cm^2^ culture flasks (for transcription analysis). Biofilm cultures were harvested after 5 days (t1) or 7 days (t2).

### Scanning electron microscopy

*M. abscessus* biofilms were grown as described above. Using 70% ethanol-sterilized microscopy cover slips and tweezers, the pellicles were collected from the wells and transferred to a new 24-well plate where they were fixed overnight at 4 °C with freshly prepared 2% glutaraldehyde in PBS. Afterwards, samples were prepared using two methods: airdrying or gradient alcohol dehydration. Airdrying: samples were left to airdry on an absorbent tissue. Gradient alcohol dehydration: samples were dehydrated through a gradient of ethanol solutions (25%, 50%, 75% and 100%) and then airdried. All samples were mounted on SEM stubs, coated with gold and examined in Philips XL-30 (LaB6) with Link Isis EDS.

### Confocal microscopy

*M. abscessus* electrocompetent cells (grown to OD_600_ 0.8) were transformed with pMV306_eGFP plasmid containing a zeocin resistance cassette (2.5 kV, 25 mF, 1000 O, 1 mm cuvettes) and selected on 25 µg/ml zeocin. *M. abscessus* eGFP strain was used for all florescence microscopy experiments. Pellicles were fixed with 4% paraformaldehyde for 30 min. Samples were stained with Nile Red (1 µM, 30 min), Propidium iodide (15 µM, 30 min), Alexa Flour 647 conjugated to Concanavalin A (100 µg/ml, 45 min) and FilmTracer™ SYPRO™ Ruby Biofilm Matrix Stain (10 µg/ml, 45 min). Cells were visualized on TIRF Nikon A1R system with a Ti microscope frame and a 100x/1.4 PlanApo objective. For co-localization analysis and calculation of biovolume, five images selected at random were taken from five distinct biological samples.

### Image processing

The data was analyzed using the Icy platform (http://icy.bioimageanalysis.org) (De Chaumont et al., 2012). Protocols plug-in was used to create automated pipelines for each of the analysis, using a similar method as Vega-Dominguez et al., 2020, Pike et al., 2017. Images were first denoised using a Gaussian filter and then the threshold was calculated using the Li method through the Thresholder plug-in. Images were binarized to create a region of interest (ROI) from which a signal was extracted for co-localization analysis using the Co-localization Studio plug-in (http://icy.bioimageanalysis.org/plugin/colocalization-studio/) (Lagache et al., 2015). The outputs were Pearson’s and Mander’s coefficient. The ROI Statistics plug-in was additionally used to determine the biovolume (http://icy.bioimageanalysis.org/plugin/roi-statistics/).

### RNA extraction

Total RNA was extracted from the cultures from five different experiments. 600 µl of freshly prepared lysozyme (5 mg/mL in TE pH8.0) was added to ~200 µl of pellet to digest the bacterial cell wall. Mixture was transferred to a bead beating tube and incubated at 37 °C for 30 min. Subsequently 1/10th of 10% SDS, 3 M NaCO_3_ (pH 5.2) and 720 µl acid Phenol (pH 4.2) were sequentially added with 2 min bead beating steps in-between and finally inverted for 1 min at the end. Samples were incubated at 65 °C for 5 min and mixed by inverting every 30 sec. Following a 5 min centrifugation at 14000 rpm, aqueous phase was removed to a fresh tube, topped with 600 µl of acid Phenol (pH 4.2) and mixed by inverting. The centrifugation step was repeated, and the aqueous phase topped with 500 µl of chloroform: isoamyl alcohol. The same centrifugation step was repeated and 1/10th volume of NaCO_3_, 3 volumes of 100% ethanol and 1 µl of glycogen were added, mixed well and left to precipitate overnight at −20 °C. The precipitate was washed once with 70% ethanol, airdried and resuspended in 30 µl of RNase-free H_2_O. Concentration of RNA was checked on Thermo Scientific™ *NanoDrop*™ and the quality with Agilent 2100 BioAnalyzer™ System using the appropriate cassette (Agilent RNA 6000 Nano Kit). 10 µg of isolated RNA was treated with DNase for 30 min at 37 °C and ethanol precipitated overnight at −20 °C. Precipitate was resuspended in 30 µl of water, and the concentration and quality checked as before. For removal of ribosomal RNA, the Ribo-Zero rRNA removal kit (Illumina) was used without deviations from the manufacturer’s instructions. The quality of RNA was checked on a BioAnalyzer Nano chip (Agilent). TruSeq Stranded mRNA kit (Illumina) was used for subsequent steps of cDNA strand synthesis, adenylation of 3′ ends, ligation and PCR amplification. Final dsDNA product was measured on Qubit and based on this concentration samples were prepared for sequencing.

### RNA-seq analysis and differentially expressed genes

Samples were pooled and sequenced using an Illumina NextSeq Instrument. Paired-end 75 bp reads were checked for technical artifacts using Illumina default quality filtering steps. Raw FASTQ read data were processed using the R package DuffyNGS as described previously (Vignali et al., 2011). Briefly, raw reads were passed through an alignment pipeline that first filtered out unwanted rRNA transcripts and then the main genomic alignment stage against the genome. Reads were aligned to *M. abscessus* (ASM6918) with Bowtie2 (Langmead and Salzberg, 2012), using the command line option “very-sensitive.” BAM files from the genomic alignment were combined into read depth wiggle tracks that recorded both uniquely mapped and multiply mapped reads to each of the forward and reverse strands of the genome(s) at single-nucleotide resolution. Gene transcript abundance was then measured by summing total reads landing inside annotated gene boundaries, expressed as both RPKM and raw read counts. RNA-seq data (raw fastq files and read counts) have been deposited in the GEO repository under accession number GSE165352.

A panel of 5 DE tools was used to identify gene expression changes between 5-day old biofilms (Biofilm t1) samples and planktonic samples (24 h, t1) or 7-day old biofilms (Biofilm t2) samples and planktonic samples (24 h, t1). The tools included (i) RoundRobin (in-house); (ii) RankProduct (Breitling et al., 2004); (iii) significance analysis of microarrays (SAM) (Tusher et al., 2001); (iv) EdgeR (Robinson and Smyth, 2008); and (v) DESeq2 (Love et al., 2014). Each DE tool was called with appropriate default parameters and operated on the same set of transcription results, using RPKM abundance units for RoundRobin, RankProduct, and SAM and raw read count abundance units for DESeq2 and EdgeR. All 5 DE results were then synthesized, by combining gene DE rank positions across all 5 DE tools. Specifically, a gene’s rank position in all 5 results was averaged, using a generalized mean to the 1/2 power, to yield the gene’s final net rank position. Each DE tool’s explicit measurements of differential expression (fold change) and significance (*P*-value) were similarly combined via appropriate averaging (arithmetic and geometric mean, respectively). Genes with averaged absolute log2 fold change bigger than two and multiple hypothesis adjusted *P*-value < 0.01 were considered differentially expressed.

### Metabolic pathway enrichment analysis

We mapped the significantly differentially expressed genes at biofilm t1 and t2 against the most recent genome-scale metabolic network construction of *M. tuberculosis* H37Rv iEK1011 (Kavvas et al., 2018) by identifying orthologs using OrthoVenn2 (Ling Xu et al., 2019 NAR). We used the subsystem definitions outlined in iEK1011 to explore pathway usage at the network level. We identified metabolic pathways that were significantly enriched in the *M. abscessus* biofilm stages (Benjamini Hochberg corrected hypergeometric *P*-value <= 0.05). For these pathways, we calculated the average fold-change of all genes. Additionally, we performed functional enrichment analysis of *M. abscessus* DEG sets with DAVID (Huang et al., 2008 Nat. Protoc.). Only functional terms with adjusted *P*-value < 0.05 were considered as overrepresented.

### Lipid analysis

Planktonic and biofilm cultures of *M. abscessus*, grown as previously described, were labelled with ^14^C acetic acid (1 µCi/µl PekinElmer) in a ratio of 1 µl:1 ml of culture. Planktonic cultures were labelled at OD 0.8 and harvested at OD 1.2. Pellicles were labelled after 5 days, at early maturation, and harvested after 7 days, at late maturation. Cultures were harvested by centrifugation (4000 rpm, 10 min) in 10 ml glass tubes sealed with polytetrafluoroethane (Teflon®)-lined screw cap, washed with PBS and then dried at 55 °C under air flow. Apolar and polar lipid fractions were isolated as described in Besra, 1998 (Besra, 1998). Radioactive counts of ^14^C labelled samples were measured using a scintillation counter and equalized so that 25,000 counts were loaded on each thin layer chromatography (TLC) plate (silica, 6.7x6.7 cm). After being run in various solvent systems in order to separate different fractions of the isolated lipids, radioactive TLCs were exposed to Carestream BIOMAX MR film for 48 h. Lipids were separated thrice in direction I in petroleum ether (60–80):acetone (98:2, v/v) and once in direction II in toluene:acetone (95:5, v/v). Mycolic acid methyl esters (MAMEs) were isolated as described by Besra, 1998 (Besra, 1998). Subclasses of MAMEs were separated using 2D argentation TLC as described by Singh et al. (2016). 75% of the plate was dipped in 10% aqueous solution of AgNO3 and plates were dried at 90 °C for 15 mins; samples were resolved in hexane:ethyl acetate (19:1) twice in dimension I and then in petroleum ether:diethyl ether (17:3) three times in dimension II.

### Mass spectrometry

After initial characterization using ^14^C-labelled samples, cultures were up-scaled to 3 L for planktonic and 500 ml for pellicles and grown without ^14^C labelling for lipid extraction and analysis by mass spectrometry. A silica column was used to separate MAMEs using a percentage gradient of toluene:ethyl acetate. Relevant fractions were analysed by MALDI-TOF/MS using the Voyager DE-STR MALDI-TOF instrument.

## Results

### *M. abscessus* forms an organized biofilm enveloped in a potential ECM

**Fig. 1:**
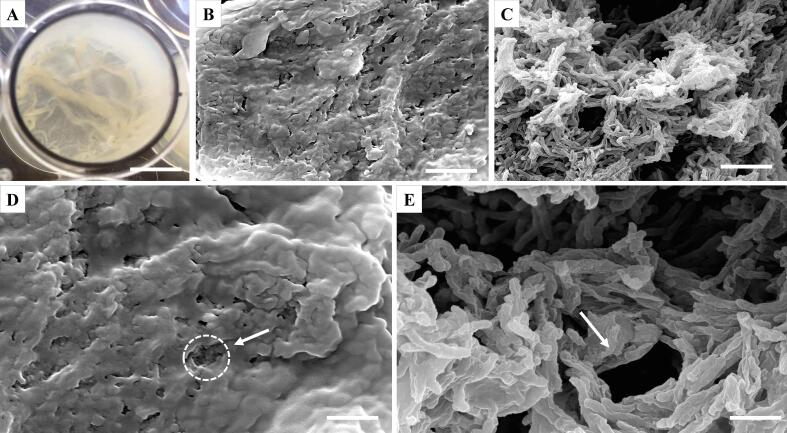
Scanning electron micrographs of *M. abscessus* pellicles grown in Sauton’s media. A. 6-day old pellicle in a 24-well plate (Φ15.6 mm, scale bar = 8 mm); B.–E. micrographs of *M. abscessus* pellicles under 5000x magnification (B. and C., scale bar = 5 µm) and 10000x magnification (D. and E., scale bar = 2 µm). Pellicles were airdried (B. and D.) or treated with gradual alcohol dehydration (C. and E.). Circle and arrows point to pores in the biofilm.

*M. abscessus* can grow as a pellicular biofilm on a liquid–air interface, that is opaque and wrinkly in appearance (Fig. 1A). Using scanning electron microscopy (SEM), we studied the formation of *M. abscessus* pellicles after a 6-day incubation. Air-dried biofilms revealed a thick substance, likely an ECM, coating the bacteria under which individual cells are not distinguishable (Fig. 1B and D). When subjected to gradual alcohol dehydration, this layer was stripped from the biofilm revealing single, stacked bacilli arranged in an organized manner allowing for formation of pores and channels, as indicated by white arrows (Fig. 1C and E).

### Fluorescent images revealed the macromolecular content of *M. abscessus* biofilms

SEM studies on the ECM were complemented with confocal laser scanning microscopy (CLSM) using an enhanced green fluorescence protein (eGFP) expressing *M. abscessus* strain and a set of fluorophores to selectively label components of the ECM. Our previous studies with *M. chelonae* biofilms showed that these fluorophores can reveal biofilm matrix composition (Vega-Dominguez et al., 2020). Concanavalin A conjugated to Alexa Flour 647 (AF) was used for staining carbohydrates, Propidium iodide (PI) for nucleic acids, FilmTracer™ SYPRO® Ruby (SR) for proteins and Nile red (NR) for lipids.

**Fig. 2:**
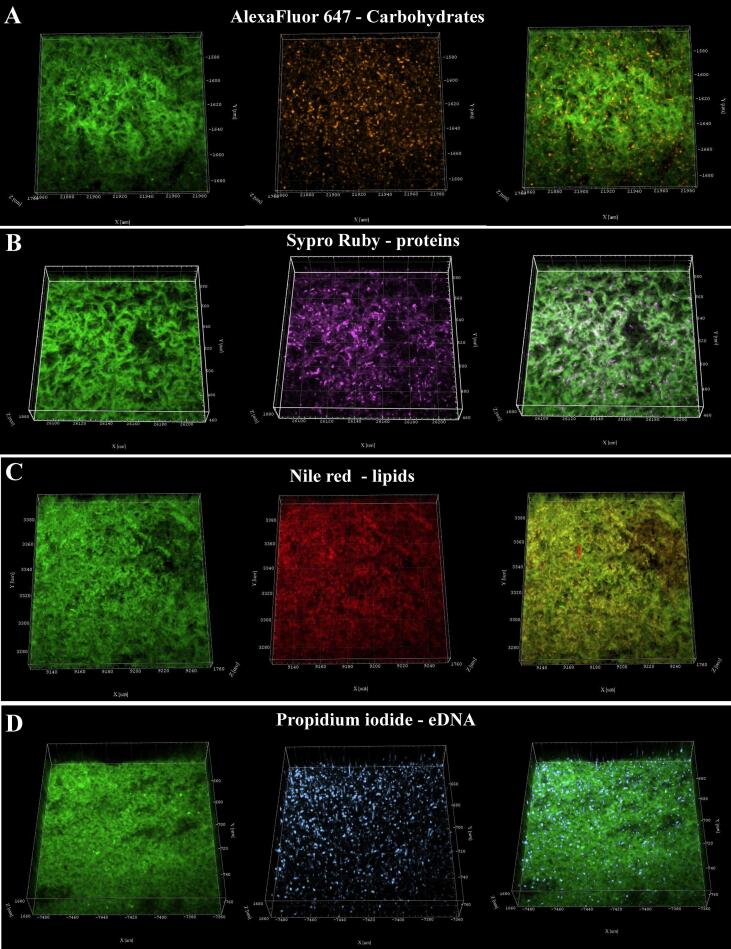
Confocal laser scanning microscopy pictures of 6-day old *M. abscessus* pellicles. 3D projections of confocal z-stacks showing in a row, left to right, eGFP-expressing *M. abscessus*, dyed component of ECM and an overlay of the two; A. Carbohydrates stained with Alexa Fluor 647 conjugated to Concanavalin A; B. Proteins stained with Sypro Ruby; C. Lipids stained with Nile Red; D. eDNA stained with Propidium iodide. (For interpretation of the references to colour in this figure legend, the reader is referred to the web version of this article.)

Confocal microscopy revealed the presence of proteins, eDNA, lipids and carbohydrates in the *M. abscessus* biofilm matrix (Fig. 2). A visual assessment of the micrographs indicated that lipids were the most abundant biomolecule in the biofilms, while proteins, eDNA, and carbohydrates were present, but more dispersed throughout the biofilm structure. Fluorescent images were quantified by calculating the relative volume of each component and evaluating co-localization of the emitted signals of GFP and each dye using Pearson’s coefficient, and co-occurrence using Mander’s coefficients (Schlafer and Meyer, 2017). Pearson’s correlation is a well-established method for presenting correlation and has been adapted to measure co-localization between fluorophores by showing the degree to which the signals linearly correlate with each other. A value of 1 (+/-) indicates a perfect positive or negative relationship, while 0 shows absence of a relationship. A high Pearson’s correlation coefficient (PCC), denoted with *r,* indicates that both signals from GFP and a fluorophore increase or decrease proportionally, while a low coefficient denotes that signal intensity of one does not alter with the change in intensity of the other.

Mander’s coefficients measure co-occurrence and were derived as an improvement to PCC since Pearson’s is not sensitive to signal intensity between different parts of an image. M1 and M2 measure the fraction of a given signal that overlaps with another signal. In this case M1 measures how the GFP signal overlaps with a signal emitted from a dye and M2 measures the opposite, how much of the dye signal overlaps with the GFP signal. A value of 1 indicates all of the signal overlaps, while 0 means none of it does. Measuring Mander’s co-efficients is an excellent way to evaluate the co-occurrence of ECM components with cells in biofilms, because it can quantify signal only present in the ECM, meaning the fluorophore signal that does not overlap with the GFP signal. The results of co-localization and biovolume analysis are summarized in Table 1.

**Table 1:** Relative biovolume of ECM components, and colocalization coefficients *r*, M1 and M2 measuring correlation and co-occurrence of eGFP signal with signals of fluorophores staining components of the ECM.

Component of ECM (dye)	Relative biovolume	Pearson’s correlation coefficient (*r*)	Mander’s coefficient	
			M1	M2
Carbohydrates (Alexa Flour 674)	0.166	0.151	0.473	0.7351
Proteins (Sypro Ruby)	0.693	0.536	0.585	0.2905
Lipids (Nile red)	1.252	0.8998	0.794	0.6872
eDNA (Propidium iodide)	0.846	0.292	0.4552	0.4540

Lipids were the most abundant biomolecule in *M. abscessus* biofilms (relative biovolume = 1.252), with a high degree of co-localization with the cells (*r =* 0.8998) and a relatively high degree of co-occurrence as indicated by the M1 and M2 (Table 1). About 30% of the NR signal did not co-occur with the GFP signal, as indicated by M2 = 0.6872. This suggested that a fraction of the lipids was found only in the ECM, additionally visible in File S1, supplementary materials which shows sectoring of lipids across the ECM and patches of the red signal that do not overlap with green. Proteins, stained with SR, had a medium degree of co-localization (*r =* 0.536) with cells, and a low signal overlap of SR with GFP (M2 = 0.2905), indicating that a high proportion of detected SR signal is coming from proteins present in the ECM, independent of cells. A sector of proteins which did not co-occur with the cells is visible towards the bottom of the binarized z-stack (File S1, supplementary materials). The PI signal, staining eDNA, showed poor intensity correlation with GFP (*r* = 0.292) and GFP and PI signals overlapped equally with each other at ~ 50% (M1 = 0.4552, M2 = 0.4540). This suggested that eDNA is dispersed throughout the biofilm, and likely present in areas with low cell count, as seen at the bottom of the biofilm in binarized z-stack images (File S1, supplementary materials). Carbohydrates were the smallest component of the *M. abscessus* biofilm ECM (relative biovolume = 0.151) and showed a low degree of co-localization with cells (*r =* 0.151). Around 70% of the AF signal overlapped with the GFP signal (M2 = 0.7351) indicating a high degree of co-occurrence, but also showing the remaining ~ 30% of AF signal occurred separately from the GFP signal and can be associated with carbohydrates in the ECM, which appear dispersed throughout (File S1, supplementary materials). In summary, lipids were the most abundant component of the *M. abscessus* biofilm ECM, while carbohydrates were the least abundant. Some of the fluorescence signals emanating from the dye-stained biomolecules were from distinct sectors with low bacterial presence indicating they are only a part of the ECM.

### *M. abscessus* biofilms show differential regulation of lipid metabolism pathways

**Fig. 3:**
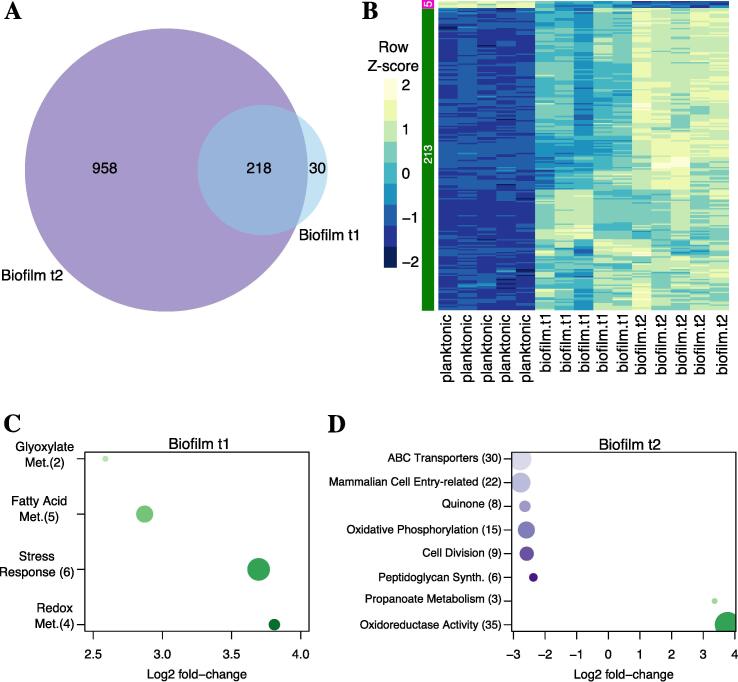
Differentially expressed genes and enriched metabolic pathways in *M. abscessus* biofilms. A. Venn diagram illustrating differentially expressed genes (DEGs) during biofilm t1 and t2 with respect to planktonic t1. B. Heatmap of transcriptional profiles (z-scores) of the 218 DEGs in biofilm t1 and t2. C. and D. Lists of enriched metabolic pathways showing log2 fold-change for biofilm t1 and t2 with respect to the planktonic t1 sample. Numbers in parentheses indicate the number of DEGs associated with each functional term. This quantity is also indicated by the size of each circle (the higher the number, the bigger the node). Down- and up-regulated functional terms are indicated with purple and green nodes, respectively. Darker colours indicate higher log2 fold-changes. (For interpretation of the references to colour in this figure legend, the reader is referred to the web version of this article.)

To further outline potential mechanisms for *M. abscessus* biofilm formation, we used RNA-Seq to sample expression profiles of the biofilms at two key timepoints. The first timepoint, t1, was taken 5 days post inoculation when the biofilm had matured and the second timepoint, t2, was taken 7 days post inoculation, when the biofilm was ready for dispersal. A mid-log planktonic culture was used as a baseline for comparison. Overall, there were 248 significantly differentially expressed genes (DEGs) identified in biofilm t1, (9-down and 239 upregulated) and 1,176 DEGs (513 down- and 663 upregulated) in biofilm t2 (absolute log2 fold-change > 2, *P*-value < 0.01). Out of those, 218 were differentially expressed at both timepoints (Fig. 3A), had the same directionality (i.e., up- or down- regulated) and were mainly upregulated, indicating commonality in genes likely to be associated with biofilm formation and maintenance. The metabolic pathways associated with the DEGs in biofilm t1 and t2 were assessed using pathway enrichment in comparison to *M. tuberculosis* metabolism from the recently updated genome-scale model iEK1011 (Kavvas et al., 2018). At biofilm maturation, t1, four pathways, related to stress response, fatty acid biosynthesis, glyoxylate biosynthesis and redox metabolism, were upregulated (Fig. 3C). Five upregulated genes involved in fatty acid biosynthesis, *MAB_2030*, *MAB_2028*, *MAB_2032*, *MAB_3354* and *MAB_3455c* (File S2, supplementary materials) are all involved in mycolic acid chain elongation including a desaturase involved in modification of the meromycolyl chain. At t2, eight pathways showed enrichment: six pathways, related to ABC transporters, quinone metabolism, oxidative phosphorylation, cell division, mammalian cell entry and peptidoglycan synthesis, were downregulated, and two pathways, related to oxidoreductase activity and propanoate metabolism, were upregulated (Fig. 3D).

### Biofilms of *M. abscessus* showed an altered mycolic acid profile

**Fig. 4:**
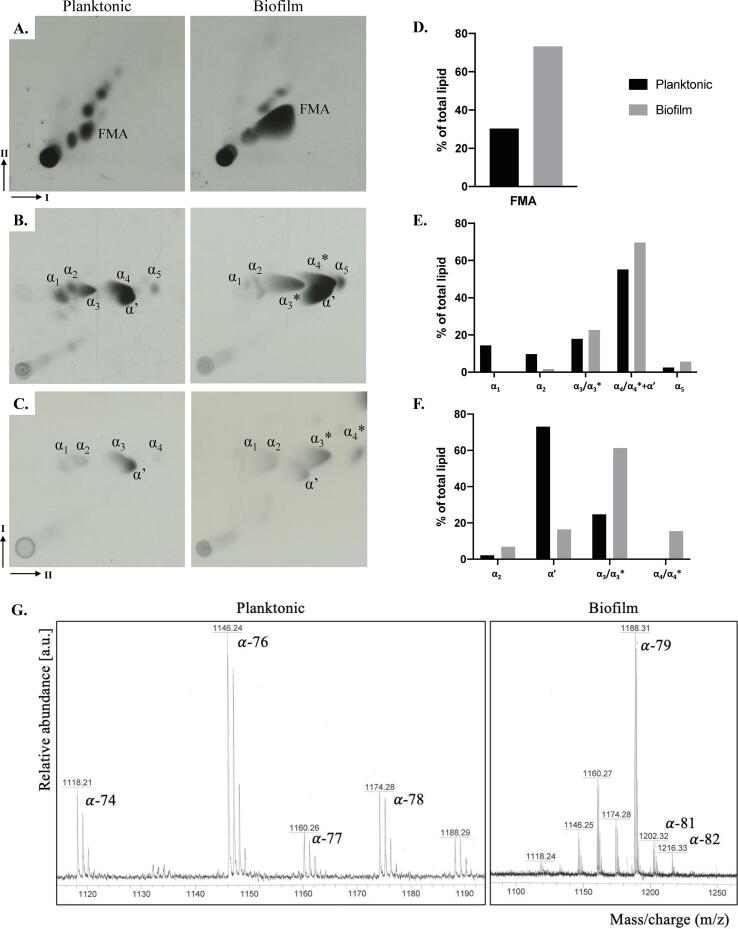
Altered expression of mycolic acids in *M. abscessus* biofilms. Autoradiographs of solvent extractable lipids separated by polarity (A–C), with bar graphs showing relative amounts of each labelled spot indicated as a percentage of total amount of lipids detected on the TLC, as determined by densitometry analysis (D–F.). A. Apolar lipids resolved using solvents in 2D dimensions (I-1st, II-2nd dimension), where FMA stands for free mycolic acids; B. and C. 2D (I-1st, II-2nd dimension) argentation TLCs of mycolic acid methyl esters (MAMEs) of wall bound (B.) and apolar lipid fractions (C.). Argentation allows for separation of molecules based on desaturation. α and α’ are mycolic acid subtypes found in *M. abscessus*, *indicates changes in species migration; G. MALDI-TOF analysis of wall bound mycolic acids annotated with C chain lengths that each molecular weight represents, more detail can be found in File S4, supplementary materials.

Based on transcription analysis that showed increase in the expression of mycolic acid biosynthesis genes in biofilms, and previous knowledge that lipids are instrumental to biofilm formation in mycobacteria (Ojha et al., 2005, Ojha et al., 2008), we did a comprehensive analysis of lipids in planktonic and biofilm *M. abscessus* (File S2, supplementary materials). Lipid fractions extracted from ^14^C supplemented cultures were resolved by thin layer chromatography (TLC) and visualized by autoradiography. The most significant difference in lipid profiles between the two growth conditions was the increase in free mycolic acids in biofilms, a phenotype observed in other mycobacteria (Ojha et al., 2008, Pacheco et al., 2013, Vega-Dominguez et al., 2020) (Fig. 4A and E). We then extracted mycolic acids as methyl esters (MAMEs) and analysed them by 2D argentated TLC to separate individual mycolic acid subspecies based on degree of desaturation (increased number of double bonds results in retardation in the second AgNO_3_-containing dimension) (Fig. 4 B and C). We observed a qualitative difference in the patterns of migration of α MAMEs. Rapidly migrating α MAMEs, labelled as α_3_* and α_4_*, were observed in biofilms (Fig. 4B and C) and were absent in the planktonic cultures, but replaced by slower migrating species, α_3_ and α_4_. Matrix-assisted laser desorption/ionization-time of flight (MALDI-TOF) analysis of the extracted MAMEs did not show any qualitative differences between biofilm and planktonic cultures, except that those extracted from biofilms were slightly longer compared to those from planktonic cultures (Fig. 4G and File S4, supplementary materials). The concurrent replacement of α_3_ and α_4_ MAMEs with the relatively faster migrating α_3_* and α_4_* suggested that α_3_* and α_4_* were likely cyclopropanated derivates of α_3_ and α4 mycolic acids. MALDI-TOF analysis would not reveal the presence of the cyclopropane rings given that these modified species would simply show a mass reflecting an additional CH_2_ unit. We observed α-MAMEs in planktonic cells that range from C-74 to C-79, with the foremost abundant species being C-76. In biofilms we observed a range from C-74 to C-82, with C-79 being the most abundant species, indicating that longer-chain MAMEs were more abundant in biofilms (Fig. 4G). From previous studies we know that in α and α’-mycolic acid structure the α-alkyl chain consists of 21 repeating units of CH_2_ with a methyl group at the end, so the variation we see are in the elongation or cyclopropanation of the β-hydroxy chain (Halloum et al., 2016).

## Discussion

In this study we characterized the *in vitro* formation of a pellicular biofilm in *M. abscessus*, describing the appearance and contents of the ECM and determining its unique transcriptional profile with a particular focus on the impact on changes in mycolic acid expression. We visualized the ubiquitous pattern of cellular organization, where cells enveloped in an ECM were aggregated in an organized manner forming channels and pores that are also visible through the ECM (Fig. 1). Using CLSM, we showed that the ECM consists of lipids, polysaccharides, proteins and eDNA (Fig. 2), all previously reported to play integral roles in biofilm formation and maturation in various bacterial species (Rose et al., 2015). We found that eDNA is abundant in *M. abscessus* biofilms (relative biovolume = 0.846) and dispersed throughout the biofilm, particularly in areas with low cell count which points to eDNA having a role in aggregation of the biofilm mirroring its function in surface-attached biofilms, previously outlined in *M. avium* studies (Rose et al., 2015). In contrast to the significant role carbohydrates play in *M. chelonae* and *M. tuberculosis* biofilms (Chakraborty and Kumar, 2019, Trivedi et al., 2016, Vega-Dominguez et al., 2020), in *M. abscessus* pellicles carbohydrates appear dispersed as evidenced by the trace presence in the ECM (Table 1). A study by Lemassu et al. (1996) similarly showed that surface exposed material of rapidly growing mycobacteria is protein-rich rather than carbohydrate-rich, in direct contrast to slow growing mycobacteria where the opposite is true – a correlation to the differences in the amount of carbohydrates reported in ECMs of *M. chelonae* and *M. tuberculosis* and our reports in *M. abscessus*.

In *M. abscessus* biofilms lipids were found to co-localize with cells to a high degree (*r* = 0.8998), however Mander’s coefficients showed that while a significant portion of the NR signal co-occurred with GFP signal (M2 = 0.687), and vice versa (M1 = 0.794), a portion of the signal did not overlap indicating presence of lipids in the ECM separately from the cells (Table 1, Fig. 2C). As a result of the unique make up of mycobacterial membranes, function of lipids has been thoroughly researched and several species are known constituents of the ECM, or have been shown to play an important role in biofilm formation and maintenance (Ripoll et al., 2007, Schorey and Sweet, 2008, Trivedi et al., 2016, Yamazaki et al., 2006). Deletion of genes responsible for glycopeptidolipid biosynthesis impaired biofilm formation in *M. avium* (Yamazaki et al., 2006), however analysis showed no notable difference in glycopeptidolipid expression between planktonic and biofilm growth in *M. abscessus* (data not shown) (Ripoll et al., 2007, Schorey and Sweet, 2008). An increase in phosphatidyl myo-inositol mannosides (PIMs), however, was noted (File S3, supplementary materials), which could point to a role in maintenance of biofilm structure, since altered acetylation of PIMs was shown to lead to defective biofilm formation (Li et al., 2020).

A notable outcome of this study is the visible increase in free mycolic acids in biofilms (Fig. 4), often described as a hallmark of biofilm formation in NTMs (Ojha et al., 2005, Ojha et al., 2008, Sambandan et al., 2013, Vega-Dominguez et al., 2020). α and α’-mycolic acid are the two identified mycolic acid subtypes in *M. abscessus* planktonic cells (Halloum et al., 2016). Αlpha-mycolic acids consists of a long carbon chain (C_74_-C_79_) containing two *cis/trans* double bonds or *cis*-cyclopropyl groups in the meromycolate chain, while α’-mycolic acids have a shorter carbon chain (C_62_-C_64_) and contain only a single *cis* double bond (Marrakchi et al., 2014). We report an accumulation of free mycolic acids in biofilms (Fig. 4A), and a change in α-mycolic acids indicative of an increase in chain length and a decrease in desaturation, or more likely, an increase in cyclopropanation. Importance of longer chain mycolic acids in biofilms was showed through studies in *M. smegmatis* and *Mycobacterium bovis* BCG where the deletion of genes related to mycolic acid biosynthesis, specifically those that encode KasB and GroEL1, a KasA chaperone, caused a decrease in mycolate chain length and impacted biofilm formation (Ojha et al., 2005, Gao et al., 2003, Wang et al., 2011). In *M. abscessus* biofilms we report an upregulation of the mycolic acid pathway (Fig. 3), with a specific focus on elongation and desaturation of the meromycolic chain. There is a strong upregulation of *MAB_2030* and *MAB_2028,* encoding the KasA/B complex which is responsible for elongation and *MAB_3354,* encoding DesA1, a desaturase protein responsible for addition of double bonds to the β-hydroxy branch (Singh et al., 2016). The evidence from our phenotypic study, coupled with the transcriptional analysis, shows that in *M. abscessus* biofilms there is an accumulation of long chain mycolic acids, with an initial increase in desaturated molecules that likely become cyclopropanated. Upregulation of the enzymes of the glyoxylate shunt, as seen in *M. abscessus* biofilm t1 (Fig. 3C), was previously reported in nutrient starvation, hypoxia, and in *M. tuberculosis* persister cells (Giffin et al., 2016, Miranda-CasoLuengo et al., 2016). Isocitrate lyase, *MAB_4095c,* which is upregulated as part of this pathway, is necessary for establishment of chronic infections with *M. tuberculosis* (McKinney et al., 2000), and inhibition of this enzyme forces bacteria to re-enter a replicative stage, implying its importance in dormancy and biofilms (Van Schaik et al., 2009). Together the upregulation of glyoxylate and fatty acid metabolism was shown to allow bacteria to survive in low nutrient environments in biofilms and to modify the cell surface and decrease susceptibility to antibiotics (Rojony et al., 2020, Rojony et al., 2019), an event we are likely registering in *M. abscessus* biofilms as well. Upregulation of the redox metabolism and stress response in biofilm t1 (Fig. 3C), and oxidoreductases in biofilm t2, indicates an adaptation of the cells to the new biofilm environment or that certain parts of the biofilm are not receiving enough oxygen. In *M. smegmatis* the ratio of NADH/NAD^+^ is three-fold higher in biofilms than in planktonic cells representative of a reductive environment, and fluctuations in the redox state of the bacilli and (p)ppGpp and c-di-GMP, markers of stress response, play critical roles in biofilm formation (Geier et al., 2008, Gupta et al., 2015, Trivedi et al., 2016, Wolff et al., 2015). Upregulated genes in these pathways mainly encode for subunits of cytochrome *d* ubiquinol oxidases, CydA, CydB and CydD, all shown to be necessary in *Escherichia coli* to form a mature biofilm and an ECM (Beebout et al., 2019). Additionally, we found genes responsible for mammalian cell entry to be downregulated in the biofilm late stage (t2) (Fig. 3D) similarly to reports in *M. chelonae* biofilms (Vega-Dominguez et al., 2020) but contrary to those in *M. tuberculosis* where *mce* operons are upregulated in stationary phase (Singh et al., 2016). Downregulation of genes encoding for mammalian cell entry proteins and for ABC transporters could be linked to the increase in mycolic acids in *M. abscessus* biofilms (Fig. 4A), given that *mce1* encodes 13 genes responsible for lipid transport and that the accumulation of mycolic acids was previously seen in Δ*mce1 M. tuberculosis* (Cantrell et al., 2013).

In conclusion, the transition from planktonic to biofilm growth in *M. abscessus* induces a significant metabolic change reflected in the phenotype. *M. abscessus* biofilm ECM is composed of carbohydrates, lipids, proteins and eDNA, with lipids making up the biggest component and mycolic acids specifically undergoing molecular changes traceable in the transcription profile. Upregulation of genes that encode for elongation and desaturation of α-mycolates is reflected in the shift to longer carbon chains in biofilm mycolic acids. The upregulation of ofglyoxylate metabolism and isocitrate lyase and the downregulation of cell division and peptidoglycan biosynthesis in biofilms are indicative of an environment of energy preservation, while the increase of redox metabolism and oxidoreductases suggest an anaerobic environment in parts of the biofilm. In fact, the CF lung environment, in which *M. abscessus* forms biofilm-like aggregates, is known to have hypoxic conditions and it was shown that *in vitro* bacterial aggregation leads to a decrease in O_2_ consumption leading to slow growth, low aerobic respiration and an increased resistance to aminoglycosides (Kolpen et al., 2020). Overall, molecular changes that occur in the *M. abscessus* biofilms promote survival, protection and modulation of the cell envelope. Rough variants of *M. abscessus* also form pellicular biofilms, and it would be interesting to see if pellicles of rough strains produce an ECM, and if so, whether they vary in composition and structure. This initial characterization of the *M. abscessus* pellicle gives a firm stepping stone for further studies into clinical treatments for dispersion of *M. abscessus* biofilms.

## CRediT authorship contribution statement

**Anja Dokic:** Conceptualization, Investigation, Visualization, Validation, Methodology, Formal analysis, Writing - original draft, Writing - review & editing. **Eliza Peterson:** Conceptualization, Investigation, Visualization, Validation, Methodology, Formal analysis, Writing - original draft, Writing - review & editing. **Mario L. Arrieta-Ortiz:** Investigation, Methodology, Formal analysis, Visualization, Writing - original draft, Writing - review & editing. **Min Pan:** Investigation, Validation, Methodology. **Alessandro Di Maio:** Methodology, Formal analysis, Writing - review & editing. **Nitin Baliga:** Conceptualization, Writing - review & editing. **Apoorva Bhatt:** Conceptualization, Writing - original draft, Writing - review & editing.

## Declaration of Competing Interest

The authors declare that they have no known competing financial interests or personal relationships that could have appeared to influence the work reported in this paper.
