# Supplementary material for: Mycobacterium abscessus biofilms produce an extracellular matrix and have a distinct mycolic acid profile
Source: Cell Surf. 2021 Apr 6;7:100051. doi: 10.1016/j.tcsw.2021.100051 (PMC8066798; doi:10.1016/j.tcsw.2021.100051)
Supplement: Supplementary data 1 [file mmc1.pptx]

## Slide 1
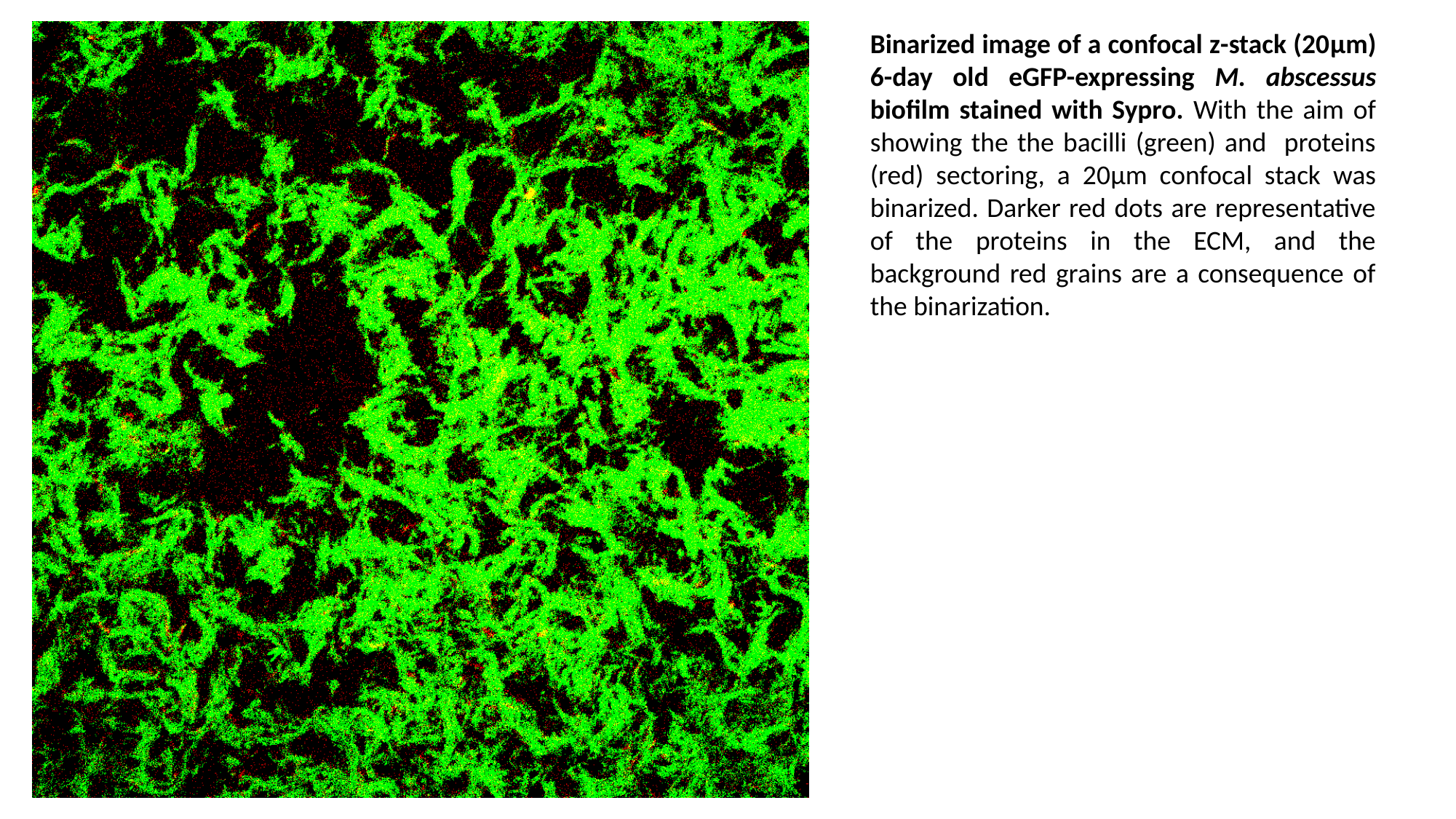

Binarized image of a confocal z-stack (20μm) 6-day old eGFP-expressing M. abscessus biofilm stained with Sypro. With the aim of showing the the bacilli (green) and proteins (red) sectoring, a 20μm confocal stack was binarized. Darker red dots are representative of the proteins in the ECM, and the background red grains are a consequence of the binarization.

## Slide 2
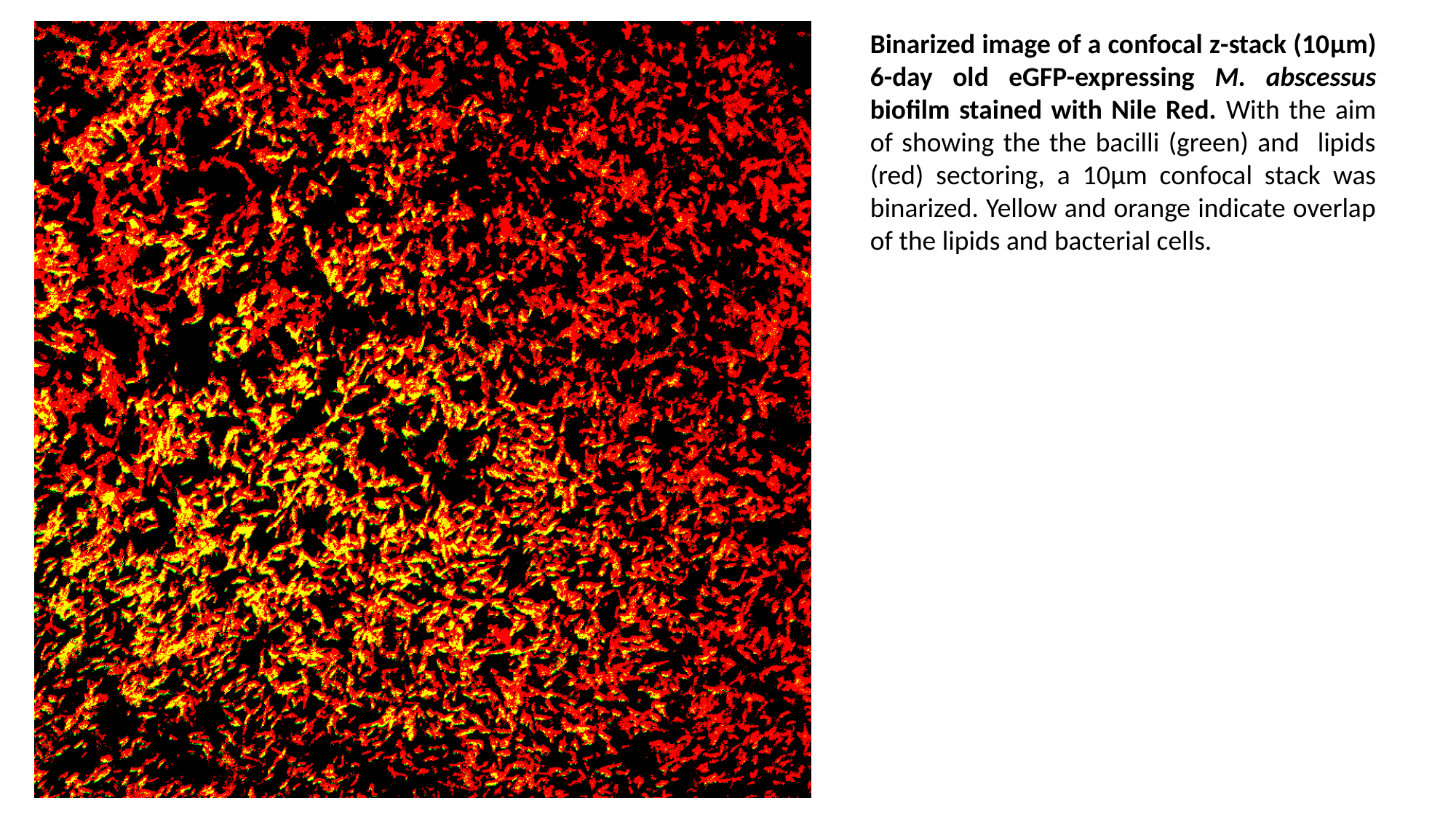

Binarized image of a confocal z-stack (10μm) 6-day old eGFP-expressing M. abscessus biofilm stained with Nile Red. With the aim of showing the the bacilli (green) and lipids (red) sectoring, a 10μm confocal stack was binarized. Yellow and orange indicate overlap of the lipids and bacterial cells.

## Slide 3
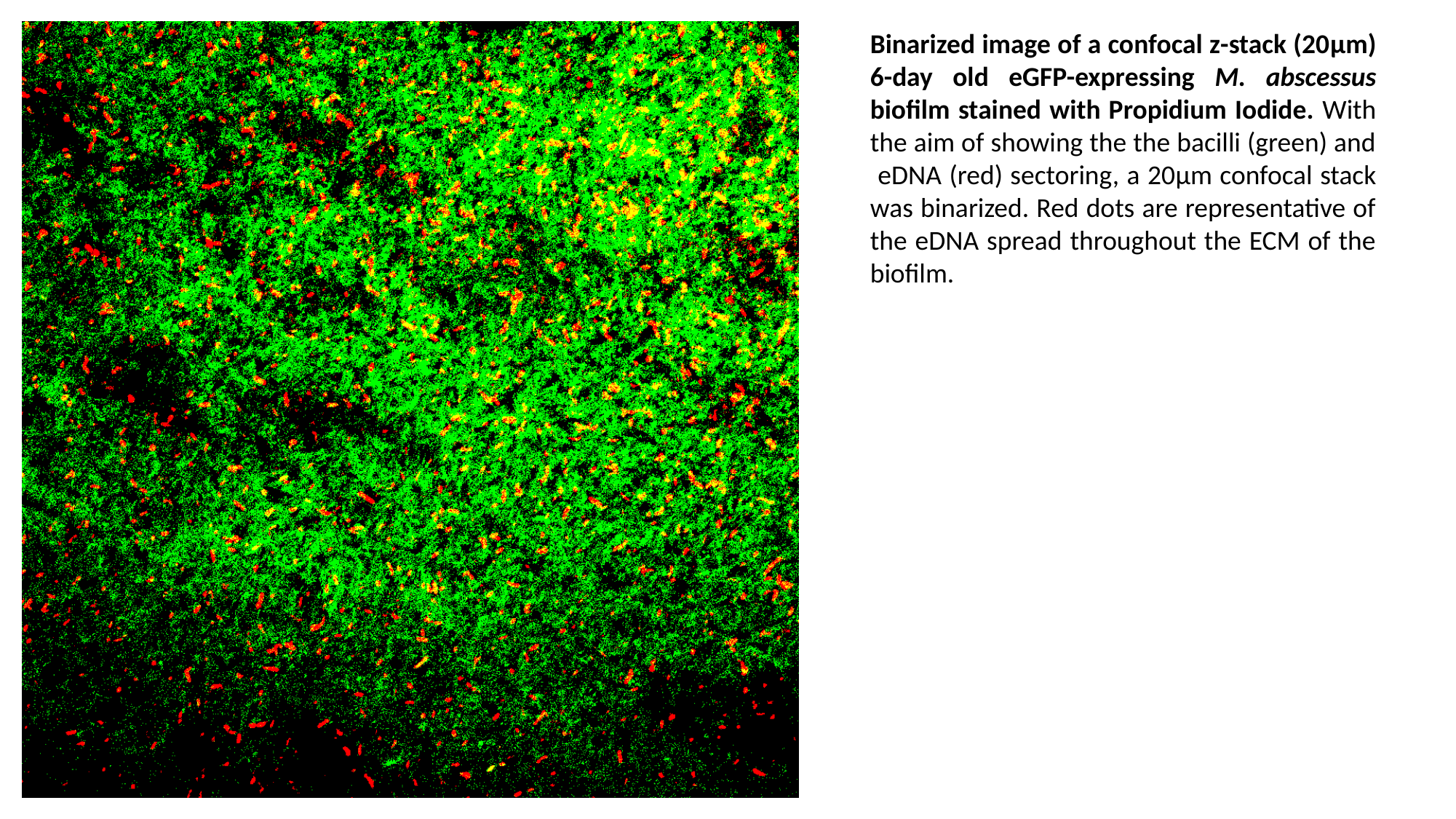

Binarized image of a confocal z-stack (20μm) 6-day old eGFP-expressing M. abscessus biofilm stained with Propidium Iodide. With the aim of showing the the bacilli (green) and eDNA (red) sectoring, a 20μm confocal stack was binarized. Red dots are representative of the eDNA spread throughout the ECM of the biofilm.

## Slide 4
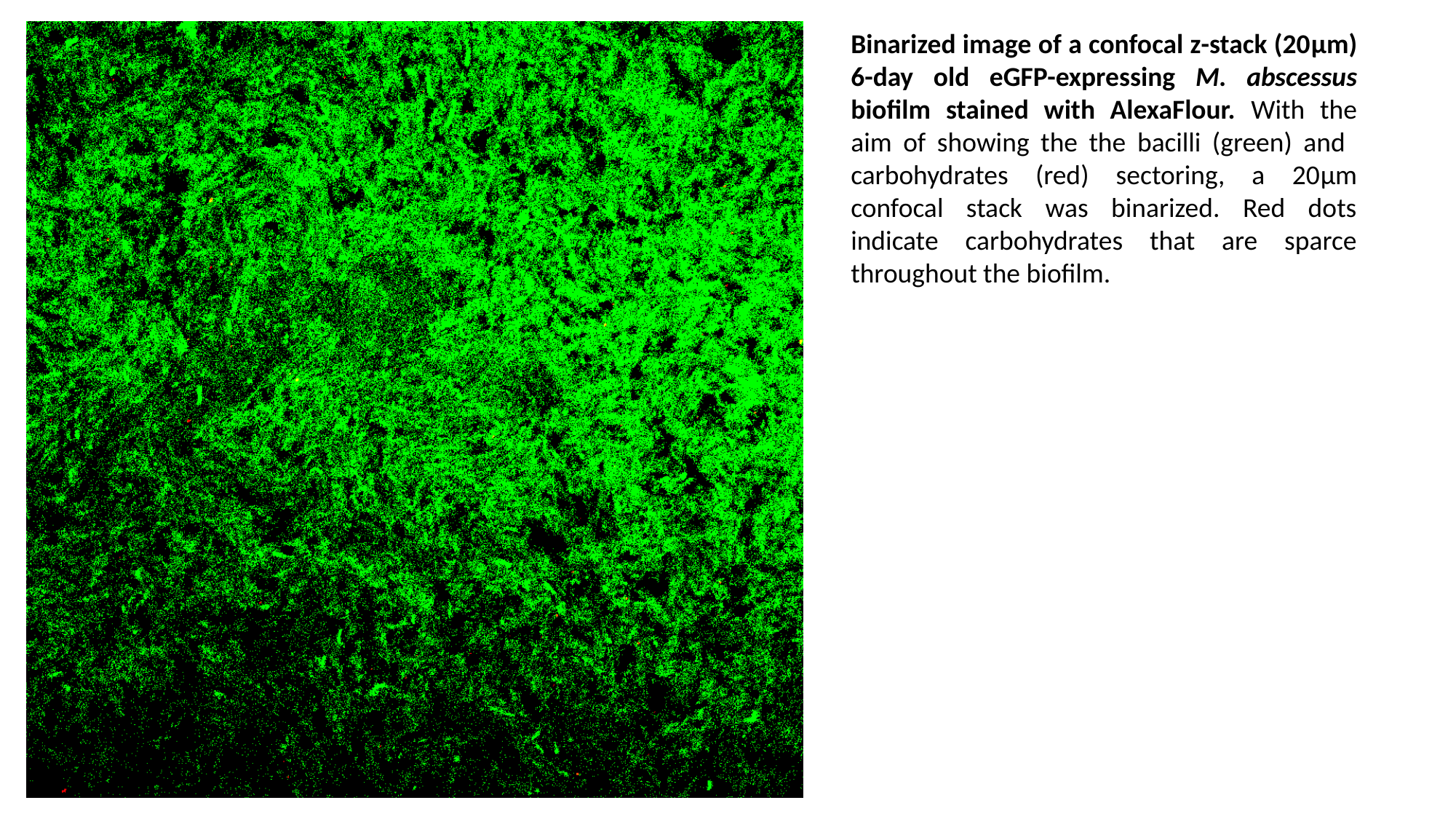

Binarized image of a confocal z-stack (20μm) 6-day old eGFP-expressing M. abscessus biofilm stained with AlexaFlour. With the aim of showing the the bacilli (green) and carbohydrates (red) sectoring, a 20μm confocal stack was binarized. Red dots indicate carbohydrates that are sparce throughout the biofilm.
