# Supplementary material for: Mycobacterium abscessus biofilms produce an extracellular matrix and have a distinct mycolic acid profile
Source: Cell Surf. 2021 Apr 6;7:100051. doi: 10.1016/j.tcsw.2021.100051 (PMC8066798; doi:10.1016/j.tcsw.2021.100051)
Supplement: Supplementary data 3 [file mmc3.docx]

**
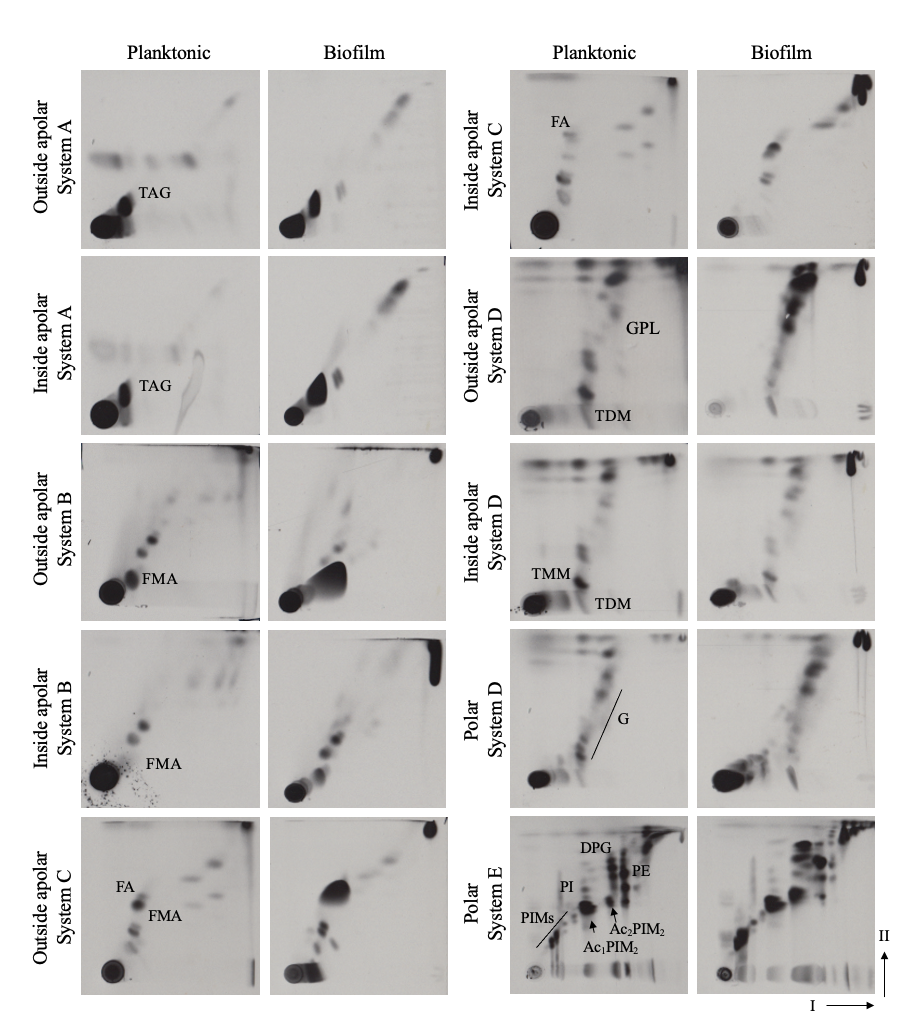
Supplementary figure 3:** Polar and apolar lipids resolved in various solvent system, listed in Besra, 1998, showing the full lipid profile of *M. abscessus* in planktonic and biofilm cells. TAG, triacylglycerols; FA, fatty acid; FMA, free mycolic acid, TMM, trehalose, monomycolate; TDM, trehalose dimycolate, G,glycolipids; P, phospholipids; DPG, diphosphatidylglycerl; PE, phosphatidylethanolamine; PI, phosphatidylinositol; PIMs, phosphatidylinositol mannosides; Ac_2_PIM_2_, diacyl phosphatidylinositol dimannoside; Ac_2_PIM_6_, diacyl phosphatidylinositol hexamannoside.
