# Supplementary material for: Mycobacterium abscessus biofilms produce an extracellular matrix and have a distinct mycolic acid profile
Source: Cell Surf. 2021 Apr 6;7:100051. doi: 10.1016/j.tcsw.2021.100051 (PMC8066798; doi:10.1016/j.tcsw.2021.100051)
Supplement: Supplementary data 4 [file mmc4.pptx]

## Slide 1
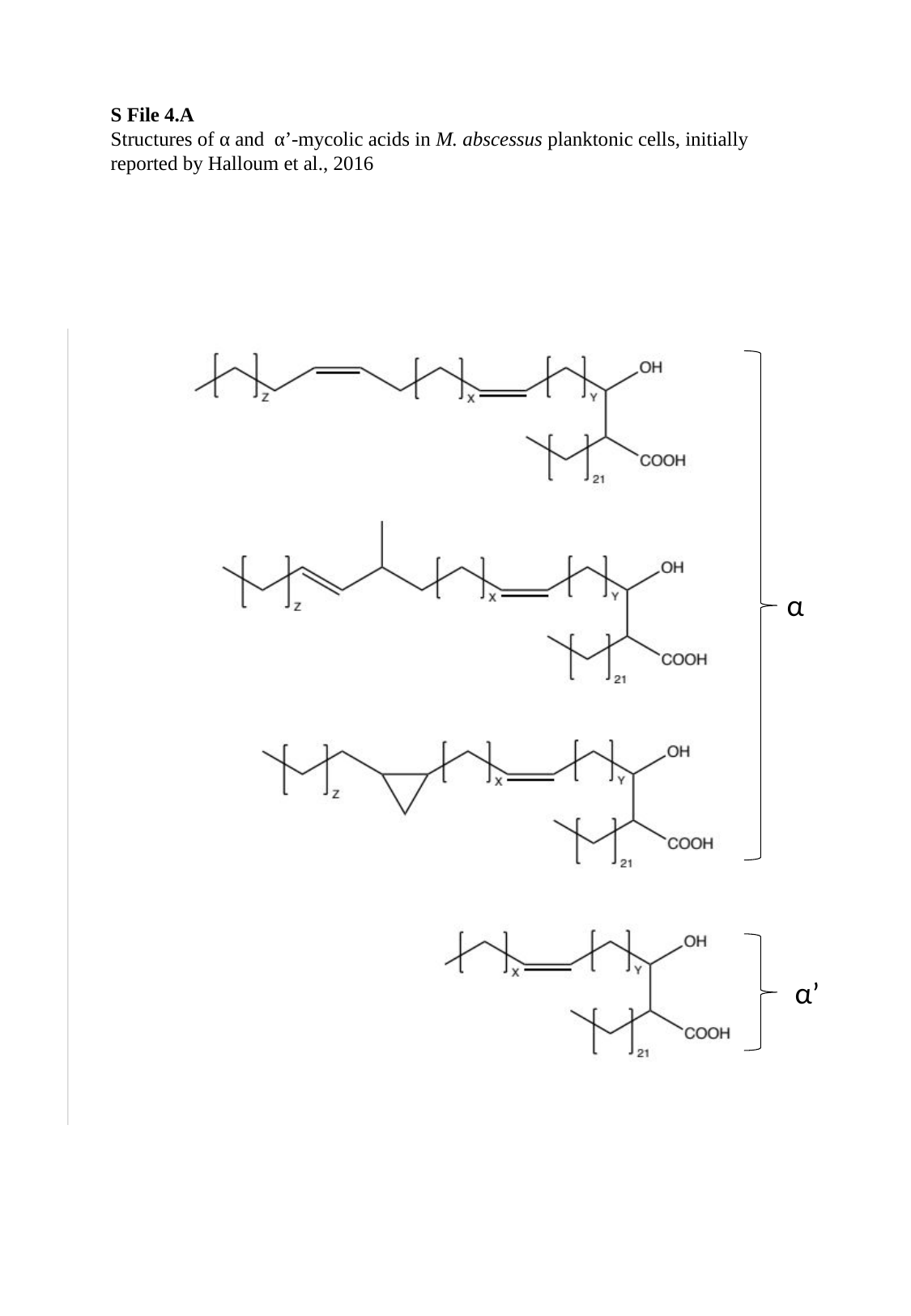

S File 4.A
Structures of α and α’-mycolic acids in M. abscessus planktonic cells, initially reported by Halloum et al., 2016
α
α’

## Slide 2
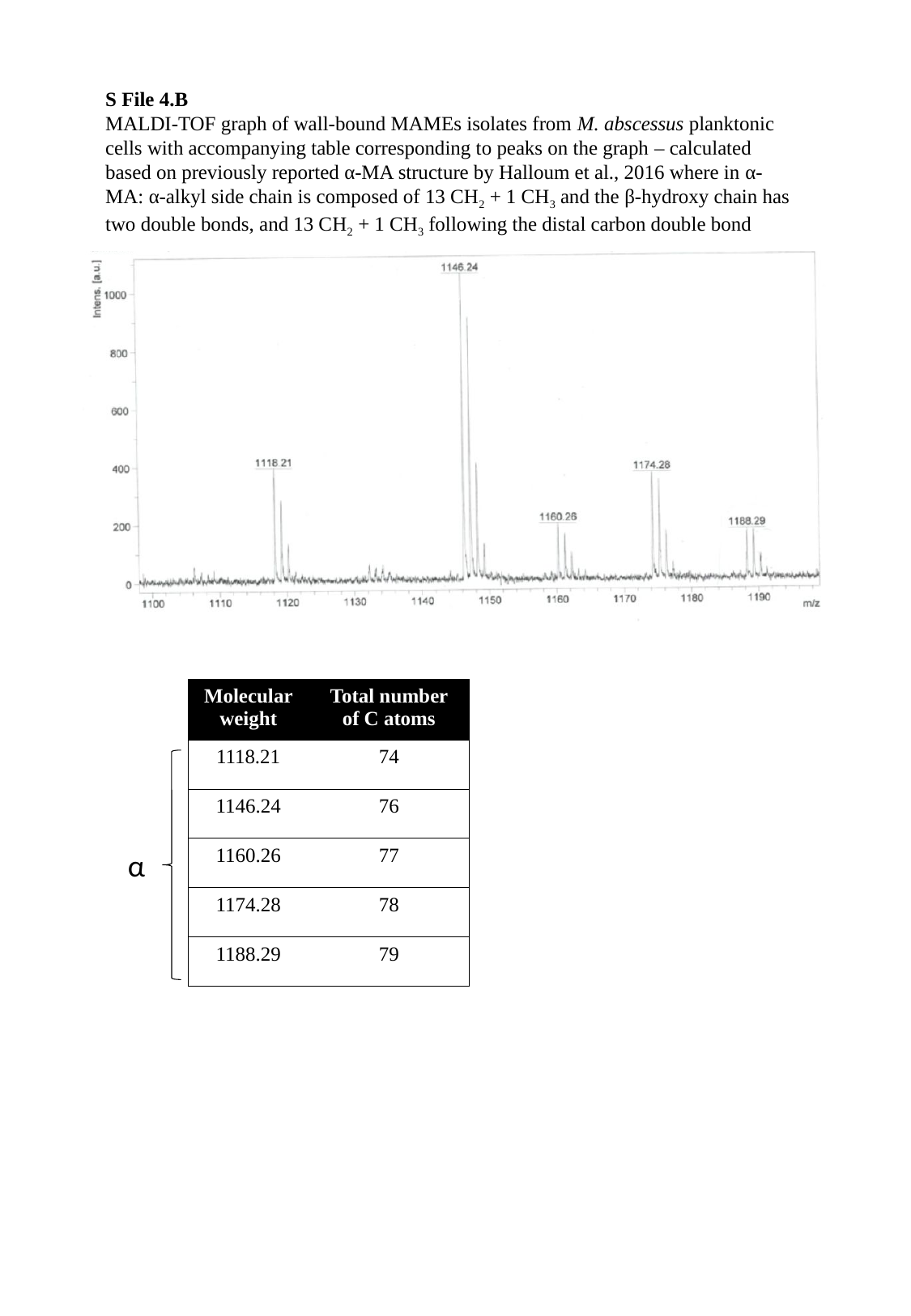

S File 4.B
MALDI-TOF graph of wall-bound MAMEs isolates from M. abscessus planktonic cells with accompanying table corresponding to peaks on the graph – calculated based on previously reported α-MA structure by Halloum et al., 2016 where in α-MA: α-alkyl side chain is composed of 13 CH2 + 1 CH3 and the β-hydroxy chain has two double bonds, and 13 CH2 + 1 CH3 following the distal carbon double bond
| Molecular weight | Total number of C atoms |
| --- | --- |
| 1118.21 | 74 |
| 1146.24 | 76 |
| 1160.26 | 77 |
| 1174.28 | 78 |
| 1188.29 | 79 |
α

## Slide 3
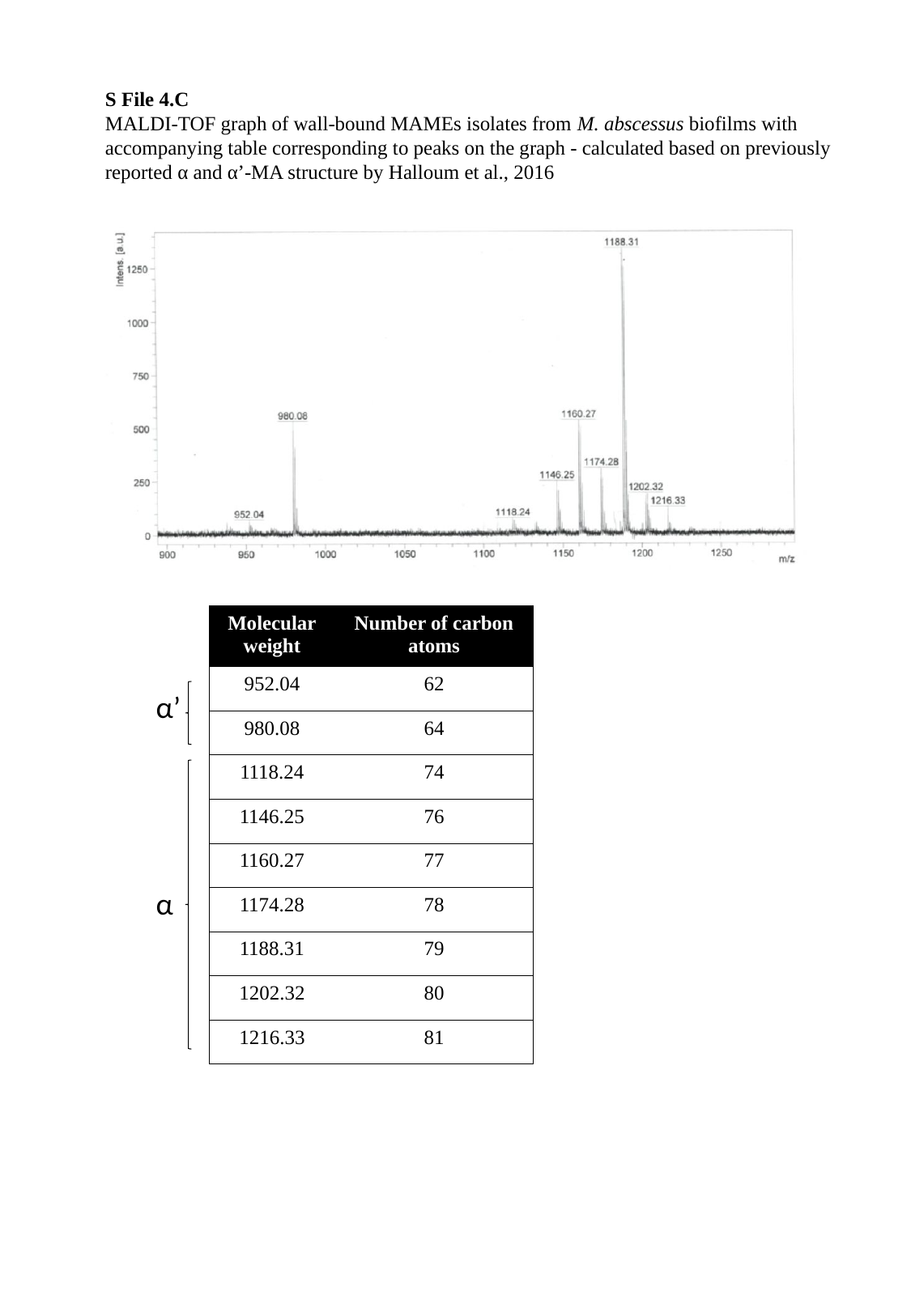

S File 4.C
MALDI-TOF graph of wall-bound MAMEs isolates from M. abscessus biofilms with accompanying table corresponding to peaks on the graph - calculated based on previously reported α and α’-MA structure by Halloum et al., 2016
| Molecular weight | Number of carbon atoms |
| --- | --- |
| 952.04 | 62 |
| 980.08 | 64 |
| 1118.24 | 74 |
| 1146.25 | 76 |
| 1160.27 | 77 |
| 1174.28 | 78 |
| 1188.31 | 79 |
| 1202.32 | 80 |
| 1216.33 | 81 |
α’
α
